# Circadian Rhythms in Regulation of Brain Processes and Role in Psychiatric Disorders

**DOI:** 10.1155/2018/5892657

**Published:** 2018-05-17

**Authors:** Harry Pantazopoulos, Karen Gamble, Oliver Stork, Shimon Amir

**Affiliations:** ^1^Translational Neuroscience Laboratory, Mclean Hospital, Belmont, MA, USA; ^2^Department of Psychiatry, Harvard Medical School, Boston, MA, USA; ^3^Department of Psychiatry and Behavioral Neurobiology, University of Alabama at Birmingham, Birmingham, AL, USA; ^4^Department of Genetics and Molecular Neurobiology, Institute of Biology, and Center for Behavioural Brain Sciences, Otto-von-Guericke University, Magdeburg, Germany; ^5^Department of Psychology, Concordia University, Montreal, QC, Canada

The molecular circadian clock regulates rhythmic transcription of thousands of genes throughout the brain and body, providing transcriptional coordination of a broad range of processes including metabolism, immune function, and DNA repair. In turn, molecular clock disruption is associated with a wide range of diseases such as heart disease, diabetes, obesity, cancer, and psychiatric disorders. Circadian rhythms in mammals are regulated by the suprachiasmatic nucleus (SCN) in the hypothalamus, which serves as the “master clock” for the brain and body. However, recent studies have revealed that clock genes are rhythmically expressed throughout the brain and play critical roles in the regulation of normal brain processes.

For example, clock genes are involved in regulating rhythms in long-term potentiation, dendritic spine regulation, receptor trafficking, and neuronal activity in brain region, and cell-type specific manners. Furthermore, recent studies have suggested a critical role of the circadian system in several disorders, including major depression, bipolar disorder, schizophrenia, anxiety, stress regulation, eating disorders, drug addiction, and alcoholism, as well as age-related cognitive deficits including Alzheimer's disease. The association of circadian rhythm disruption with cognitive function, mood, immune system disruption, and metabolism in shift workers as well as people with mood disorders provides further evidence for the effects of circadian disruption on synaptic plasticity. This research area is at the interface of neuroscience, cell biology, endocrinology, and psychiatry. Despite the evidence that circadian rhythms are involved in coordination of several neural processes and in turn behaviors, this topic has not been extensively studied thus far. There is much that is not known regarding how and why circadian rhythms regulate brain processes.

The articles in this special issue represent a multidisciplinary collection of recent advances in the role of the circadian system in normal brain functions and psychiatric disorders. They illustrate the continuing effort to understand the role of the circadian system in the regulation of normal brain processes and in diseases states, as well as the potential to take advantage of circadian regulation as a tool for the development of therapeutic strategies.

A fundamental aspect of circadian rhythm regulation is the entrainment of the SCN molecular clock by light. In the article “Photoperiodic Programming of the SCN and its Role in Photoperiodic Output,” M. C. Tackenberg and D. G. McMahon provide an overview of the mechanisms involved in entrainment of SCN rhythms and effects on potential SCN output signals, ranging from initial research on SCN entrainment to modern advances including key studies from their research group. This review serves as a foundation for understanding how the circadian system is regulated by light, explores the relevance of circadian entrainment to human health, and highlights the most important outstanding questions.

Changes in day length have long been associated with alterations in mood. Understanding how seasonal changes in light cycles impact the circuitry involved in mood regulation is critical for development of effective preventative and treatment measures. The review article by A. Porcu et al. titled “Photoperiod-Induced Neuroplasticity in the Circadian System” explores how environmental light exposure impacts clock gene and neurotransmitter expression in the SCN, as well as brain regions involved in the regulation of mood, sleep, and motivational states, and thus provides a framework of how seasonal changes in day length may translate to specific molecules and neurocircuits involved in mood regulation.

Our understanding of the roles that glial cells play in a range of normal brain processes is a rapidly expanding field in neuroscience with broad clinical implications. The article “The Role of Mammalian Glial Cells in Circadian Rhythm Regulation” by D. Chi-Castaneda and A. Ortega reviews the emerging evidence for circadian rhythms in glial cells. The existence and potential role of molecular clock rhythms in glial cells is summarized, including roles in processes with clinical implications such as glutamate reuptake, synaptic plasticity, and immune function.

Growing evidence suggests that clock genes in brain regions other than the SCN are critically involved in regulating the timing of cellular signaling during information processing and memory formation. Two review articles highlight the importance of this function: the article “Circadian Regulation of Hippocampal-Dependent Memory: Circuits, Synapses, and Molecular Mechanisms” by K. H. Snider et al. reviews the current hypotheses on the regulation of memory processing by the molecular circadian clock, with a focus on ERK/MAPK and GSK3*β*. Furthermore, they address outstanding questions in the field while examining broader implications of circadian regulation of synaptic plasticity. The article “Clocking In Time to Gate Memory Processes: The Circadian Clock Is Part of the Ins and Outs of Memory” by O. Rawashdeh et al. summarizes pioneering studies from their group and others that have accumulated evidence for the key role of circadian Per1 regulation of MAPK, cAMP, and CREB during hippocampus-dependent memory processes.

The importance of developing improved preventative and therapeutic strategies for addiction has come to the forefront recently due to the growing opioid crisis and the obesity epidemic. The next two articles highlight the involvement of the circadian system in reward and addiction. The paper “Neural Mechanisms of Circadian Regulation of Natural and Drug Reward” by L. M. DePoy et al. provides an extensive review of the current hypotheses on how the circadian system is involved in reward regulation, ranging from rhythms of reward sensitivity to effects of sleep disturbances on reward processing and in turn effects of drug addiction on the circadian system. The paper focuses on key evidence of molecular clock regulation of the dopamine system in reward processes identified by their research group. The authors thus provide a framework to help address the question of how the circadian system can be used for the treatment of addiction.

A. K. Nobrega and L. C. Lyons (“*Drosophila*: An Emergent Model for Delineating Interactions between the Circadian Clock and Drugs of Abuse”) highlight the potential of Drosophila as a model organism for identification of the complex interactions between the circadian system and addiction. This extensive review discusses the evidence for association of circadian disruption with addiction and stress in humans and highlights how the highly conserved mechanisms involved in these systems can be used in simple organisms to address key questions regarding the complex interactions of sleep, circadian rhythms, stress, and addiction. The potential of Drosophila as a model system in circadian rhythm research is exemplified by the Nobel Prize awarded to the characterization of the molecular circadian clock. The authors include a detailed summary of how this model system can be leveraged to develop novel therapeutic pharmacological targets for addiction.

Stress signaling is intricately involved with the circadian system, and the reciprocal interactions between these systems are critical for the maintenance of physiological homeostasis. Disruption of this interaction contributes to a wide array of health issues from mood disorders, cognitive dysfunction, metabolic disorders, and immune system dysfunction. Stress during critical periods of development is also believed to contribute to many of these disorders later in life. The article “Perinatal Programming of Circadian Clock-Stress Crosstalk” by M. Astiz and H. Oster reviews the current evidence for the effects of perinatal stress on the developing circadian system and the long-term health implications of these effects, potentially impacting impulsivity, stress regulation, metabolism and mood in adults.

The paper “Circadian Rhythms in Fear Conditioning: An Overview of Behavioral, Brain System, and Molecular Interactions” by A. Albrecht and O. Stork describes neural circuits involved in circadian regulation of fear memory and stress response, including recent evidence from their research group and others regarding the role of clock genes in brain areas involved in fear memory processing. The authors also discuss how the interaction of the circadian system with fear memory processing may be related to the development of psychiatric disorders characterized by excessive fear memory, including PTSD, and how this interaction may be leveraged for therapeutic purposes.

Moreover, C. A. Vadnie and C. A. McClung, in “Circadian Rhythm Disturbances in Mood Disorders: Insights into the Role of the Suprachiasmatic Nucleus,” review the considerable evidence for the involvement of circadian rhythm dysfunction in mood disorders, ranging from clinical studies to animal models, including the clock delta 19 mutant mouse model established by this group, which models several critical aspects of bipolar disorder and provides insight into potential mechanisms involved in mania. Their article examines relationships of pharmacological therapies with the circadian system and focuses on the potential role of the SCN in the etiology of mood disorders.

We conclude this issue with a paper from S. Kaladchibachi and F. Fernandez (“Precision Light for the Treatment of Psychiatric Disorders”) discussing the potential of light therapy for the treatment of psychiatric disorders. The authors provide a chronologic review of the history of studies focused on the effects of light on synchronizing the human circadian system and in the treatment of depression. They include a comparison of the effects of varying wavelengths, intensity, and duration of light exposure and propose a framework for the therapeutic use of light exposure.

We believe that the articles highlighted in this special issue provide a comprehensive overview of the current state of research on the role of the circadian system in the regulation of normal brain processes and hope that they will stimulate further studies into the circadian system contribution to cognitive, affective, and neurodegenerative brain disorders ranging from cognitive dysfunction, memory impairment, PTSD, major depression to bipolar disorder. A deeper understanding of the role the circadian system plays in brain function has the potential to allow us to leverage this system for preventative and therapeutic treatments.

*Harry Pantazopoulos* *Karen Gamble* *Oliver Stork* *Shimon Amir*

